# There are more things in physical function and pain: a systematic review on physical, mental and social health within the orthopedic fracture population using PROMIS

**DOI:** 10.1186/s41687-022-00440-3

**Published:** 2022-04-06

**Authors:** Thymen Houwen, Leonie de Munter, Koen W. W. Lansink, Mariska A. C. de Jongh

**Affiliations:** 1 Network Emergency Care Brabant, ETZ Hospital (Elisabeth-TweeSteden Ziekenhuis), Hilvarenbeekseweg 60, 5022 GC Tilburg, The Netherlands; 2 Trauma Research Unit, Department of Surgery, Erasmus MC, University Medical Center Rotterdam, P.O. Box 2040, 3000 CA Rotterdam, The Netherlands; 3 Department of Traumatology, ETZ Hospital (Elisabeth-TweeSteden Ziekenhuis), Hilvarenbeekseweg 60, 5022 GC Tilburg, the Netherlands; 4 Department of Surgery, ETZ Hospital (Elisabeth-TweeSteden Ziekenhuis), Hilvarenbeekseweg 60, 5022 GC Tilburg, the Netherlands

**Keywords:** PROMIS®, Fracture, Orthopedics, Patient-reported outcomes, Trauma

## Abstract

**Background:**

The Patient-Reported Outcomes Information System (PROMIS®) is more and more extensively being used in medical literature in patients with an orthopedic fracture. Yet, many articles studied heterogeneous groups with chronic orthopedic disorders in which fracture patients were included as well. At this moment, there is no systematic overview of the exact use of PROMIS measures in the orthopedic fracture population. Therefore this review aimed to provide an overview of the PROMIS health domains physical health, mental health and social health in patients suffering an orthopedic fracture.

**Methods:**

This systematic review was documented according to the Preferred Reporting Items for Systematic Review and Meta-Analyses (PRISMA) guidelines. We searched Embase, Medline, Web of Science Core Collection, and Cochrane Central Register of controlled Trials, CINAHL and Google Scholar in December 2020 using a combination of MeSH terms and specific index terms related to orthopedic fractures and PROMIS questionnaires. Inclusion criteria were available full text articles that were describing the use of any PROMIS questionnaires in both the adult and pediatric extremity fracture population.

**Results:**

We included 51 relevant articles of which most were observational studies (n = 47, 92.2%). A single fracture population was included in 47 studies of which 9 involved ankle fractures (9/51; 17.6%), followed by humeral fractures (8/51; 15.7%), tibia fractures (6/51; 11.8%) and radial -or ulnar fractures (5/51; 9.8%). PROMIS Physical Function (n = 32, 32/51 = 62.7%) and PROMIS Pain Interference (n = 21, 21/51 = 41.2%) were most frequently used questionnaires. PROMIS measures concerning social (n = 5/51 = 9.8%) and mental health (10/51 = 19.6%) were much less often used as outcome measures in the fracture population. A gradually increasing use of PROMIS questionnaires in the orthopedic fracture population was seen since 2017.

**Conclusion:**

Many different PROMIS measures on multiple domains are available and used in previous articles with orthopedic fracture patients. With physical function and pain interference as most popular PROMIS measures, it is important to emphasize that other health-domains such as mental and social health can also be essential to fracture patients.

**Supplementary Information:**

The online version contains supplementary material available at 10.1186/s41687-022-00440-3.

## Introduction

The number of orthopedic fractures are globally increasing and present a sincere burden on both health related and socioeconomic status of individual patients and communities [1]. Orthopedic fractures are known for negative interference in work status, health related quality of life, impairments and health care costs [1–3]. An orthopedic trauma is defined as any injury to the bones, joints and/or soft tissue caused by an external force and bone fractures are all disruptions in bone continuity. In order to measure patient outcomes, patient-reported outcomes measures (PROMs) have been evolving rapidly [4]. In clinical practice, PROMs are used to obtain patient reported information on diverse and essential health aspects like physical function, pain, and mental and social health. More specifically, the impact of treatment and health condition can be assessed by using PROMs [5, 6]**.** Many available PROMs for the orthopedic trauma populations are anatomical region specific fixed scales (e.g. FAAM, FADI, DASH, ASES or KOOS), rather than more generic questionnaires and a full survey must be completed by the patient [7, 8]. This can be time-consuming for the patient and healthcare provider, but is required to gain a valid score [9]. Short form fixed scales are also available, but might be limited in precision if the number of questions is not reduced properly [10]. On top of this, fixed scales are prone to floor and ceiling effects, since fixed scale questions are often limited of scope with regards to important health domains [11].

Given these limitations, the Patient Reported Outcomes Information System (PROMIS®) was established. PROMIS was developed to gain generic outcome measures into a more valid, generalizable and reliable method [12]. The great advantage and characteristic entity of PROMIS is the possibility of applying Computerized Adaptive Testing (CAT), which is based on the Item Response Theory (IRT). In IRT, a computerized algorithm uses the previous answer to provide the next question. Additional questions follow until a valid and precise score has been reached with a standard error less than 3.0 on the common metric of a T-score. This normally results in a compact questionnaire with less time effort for the patient and a lower administrative burden [13]**.** PROMIS measures are demonstrated in T-scores that are standardized to the (U.S.) general population. These PROMIS T-scores range from 0 to 100 with a mean of 50 and a standard deviation of 10 points on the T-score metric. Greater T-scores represent more of the outcome being quantified, thus in a positive context, a higher T-score means a better outcome and in a negative context, a higher T-score means a worse outcome.

PROMIS captures three essential health domains for a generic population, namely the physical health domain, the social health domain and the mental health domain. These three health domains are also important to orthopedic fracture patients and might, because of the acute health shift differ from general orthopedic populations [14, 15]. For example, depression or anxiety (mental health domain) after a traumatic fracture decrease quality of life, but also tend to limit physical progression and extend the usage of pain medication [16, 17]. On top of this, the measurement of physical function (physical health domain) shows the functional status of patients and could be used to interfere early into the recovery process when physical function is regressing. Lastly, fractures have been associated with a limitation in social interaction [18, 19]. Due to the advantages of PROMIS measures and due to the importance of monitoring the different health domains in orthopedic fracture patients, there is an increasing number of publications in recent medical literature, including some systematic reviews, using the PROMIS questionnaires in this subgroup [4, 20–24]. Multiple papers have been published in which PROMIS tools were seen in the view of outcome measures or in which PROMIS tools were validated, but many articles studied heterogeneous groups with chronic disorders including orthopedic patients in general [4, 8, 25–28]**.**

Previous systematic reviews on PROMIS in orthopedic patients primarily focused on physical function in general orthopedic populations, with the argument that PROMIS physical function has been most thoroughly studied in musculoskeletal disorders [23]. On top of this, some systematic reviews showed an underrepresentation of orthopedic trauma patients in general or orthopedic fracture patients or could only include a limited amount of studies on health domains different from physical function [4, 24]. Furthermore, one systematic review only assessed trauma patients with upper limb trauma [8].

Although the importance of health domains physical function, mental health and social health are evident, there is no recent systematic overview that provides a thoroughly outline of all PROMIS health domains in the orthopedic fracture population specified into subgroups with different upper and lower extremity fractures. Therefore, the primary goal of this review was to provide an overview of studied PROMIS health domains in patients suffering an orthopedic fracture. We aimed to determine the frequency and extensiveness of usage of available PROMIS measures. Secondly, the use of PROMIS differentiated by type of fracture was assessed to evaluate if PROMIS is more often used in specific fracture types.

## Methods

### Search strategy

This systematic review was documented according to the Preferred Reporting Items for Systematic Review and Meta-Analyses (PRISMA) guidelines [29].

The literature search was performed in December 2020 with the assistance of a professional biomedical information specialist at the Erasmus Medical center Rotterdam. Literature was extracted from the medical databases Embase, Medline, Web of Science Core Collection, Cochrane Central Register of controlled Trials, CINAHL and Google Scholar. A combination of MeSH terms and specific index terms related to orthopedic fractures and PROMIS questionnaires were used. See Additional file 1: Appendix 1 for the full search strategy.

### Study selection

Articles were eligible for inclusion when (1) any PROMIS questionnaires in both the adult and pediatric extremity fracture population were described, (2) the study was published in English or Dutch and (3) full text of the article was available. Since our goal was to provide an overview of the different PROMIS health domains in patients suffering an orthopedic fracture, and PROMIS offers the opportunity to explore health domains in adults and children, both groups were included. Lower extremity fractures were defined in our study as fractures reaching from pelvis to toes, and upper extremity fractures reached from scapula to fingers. Articles in which fracture patients were part of a large heterogeneous population and where results were not specified for fracture patients were excluded. Conference papers, abstracts, editorials, study protocols, systematic reviews, and meta-analysis were also excluded. Two reviewers (T.H. and L.M.) screened all papers based on title and abstract. Disagreement between the reviewers was resolved by discussion with a third member of the research team (M.J). Next, one reviewer (T.H) collected full texts of papers. The medical library was consulted if full texts of papers could not be extracted from the internet. These full-text papers were screened for eligibility by T.H. and L.M. Reference lists from selected papers were manually screened by two reviewers (T.H. and L.M.) to identify additional eligible papers.

**Fig.1:**
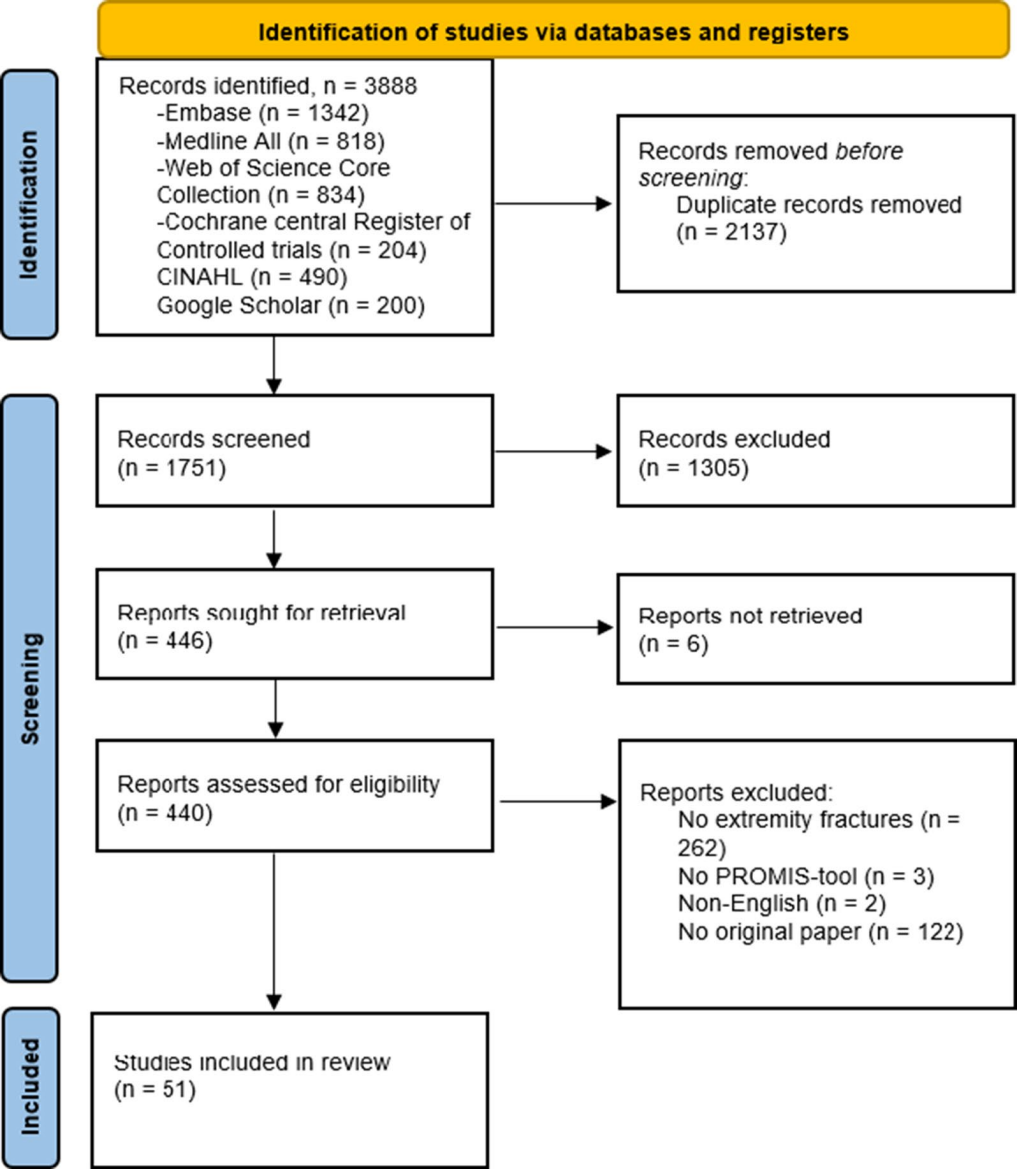
Flow diagram showing the process of identification, screening and inclusion of eligible papers

Figure 1 shows the PRISMA flow diagram illustrating the process of screening and identification of eligible articles.

### Quality assessment

Both reviewers (T.H. and L.M) assessed the quality of included observational papers by using the Strengthening. The Reporting of Observational Studies in Epidemiology (STROBE) checklist [30]. Disagreement in quality assessment was resolved by discussion with a third reviewer (M.J). The STROBE checklist provides recommendations on items that should be addressed in observational studies. Items on the STROBE checklist were reported in a binary way (1 = the item was sufficiently reported, 0 = the item was insufficiently or not reported). In total, 34 items can be scored by using the STROBE-checklist. Since the STROBE-checklist is not suitable for the assessment of case series, the CARE guidelines were used as a framework to assess content of included case series [31]. Methodological quality of randomized controlled trials was assessed by using the Downs and Black Checklist [32].

### Data extraction

The first reviewer (T.H.) extracted data from included papers onto a shared excel file, which was reviewed and if needed supplemented by the second reviewer (L.M.). Extracted information included: (1) basic study -and publication characteristics (first author, country in which the study was performed, level of evidence according Evidence-based nursing care guidelines, study design, primary aim, and inclusion and exclusion criteria); (2) study population characteristics (population type, injury type, number of patients, sex distribution, mean age, follow up time and level of trauma center); (3) PROMIS features (number of PROMIS forms, type of PROMIS forms, context and PROMIS scores).

## Results

### Study selection

The initial search resulted in the selection of 3888 papers. Duplicates were removed and 1751 articles remained eligible for screening. In total, 1305 articles were excluded following screening by title and abstract. The medical library was requested for assistance in the search for full-text papers of which 6 articles were not available, leaving 440 articles to be assessed for full text. Finally, 51 papers were included for this review.

### Overall study quality

Most articles were based on a retrospective -or prospective observational design with a cross-sectional or case–control setting (n = 47, 92.2%). Additionally, 2 (3.9%) randomized controlled trials and 2 (3.9%) case series were included.

An overview of study quality assessment can be found in Additional file 2: Appendix 2, Additional file 3: Appendix 3 and Additional file 4: Appendix 4. The mean quality score of the 47 observational articles according to the STROBE checklist was 26.5 out of 34 (SD = 3.2). More than half of the papers did describe how missing data were addressed (item 12c, n = 26 studies, 55.3%), lacked the description of any sensitivity analysis (item 12e, n = 32 studies, 68.1%) or did not use a flow diagram (item 13c, n = 39 studies, 83.0%). Papers adequately described balanced information on the scientific paper (item 1b, n = 47 studies, 100%), scientific background and rationale for the investigation (item 2, n = 47 studies, 100%), eligibility criteria (item 6a, n = 46 studies, 97.9%), key results with reference to the study objects (item 18, n = 47 studies, 100%) and limitations of the study (item 19, n = 46 studies, 97.9%). The article which was ranked as the lowest by using the STROBE checklist scored 18 points [33] versus 32 points for the article which scored the highest [34]. Two studies were assessed by using care guidelines for case series with a mean score of 22.5 out of 30 (respectively 21 and 24 points) and two studies were assessed by the Downs and Back checklist with a mean score of 20 out of 27 (respectively 18 and 22 points).

### Study characteristics

Table 1 presents the basic characteristics of the included studies.

**Table 1:** Basic characteristics of the included studies

References	Study design	Patient population	Sample size	Female percentage	Mean age in years (SD)	Mean follow-up (SD)	PROMIS scores in T scores, mean (SD)	Type of PROMIS form	Level of trauma center
Anthony et al. [43]	Randomized controlled trial	Traumatic upper or lower extremity fracture (Ankle fracture, calcaneus fracture, clavicle fracture, distal femur fracture, distal humerus fracture, elbow fracture, femoral shaft fracture, femoral neck fracture, intertrochanteric hip fracture, navicular fracture, patella fracture, polytrauma, proximal humerus fracture, sub trochanteric fracture, tibia plateau fracture, tibia plafond fracture)	82	50% (n = 41)	Acceptance and commitment therapy group 45.5 (15.9), Control group 48.7 (14.6)	NA	Acceptance and commitment therapy group, **preoperative results**: PROMIS Pain Intensity 1A Score 5.4 (2.9), PROMIS Pain Intensity 3A Score, 54.9 (7.3), PROMIS Pain Interference 8A score, 63.6 (11.4), PROMIS Emotional Distress-Anxiety 8A Score, 56.5 (11.4) **Postoperative results:** PROMIS Pain Intensity 1A Score, 3.4 (2.2), PROMIS Pain Intensity 3A Score, 45.9 (7.2) PROMIS Pain Interference 8A score, 56.6 (9.4) PROMIS Emotional Distress-Anxiety 8A, 51.5 (10.4) Control group, Preoperative **results**: PROMIS Pain Intensity 1A Score, 6.2 (2.6), PROMIS Pain Intensity 3A Score, 57.1 (8.2), PROMIS Pain Interference 8A score, 66.1 (8.4), PROMIS Emotional Distress-Anxiety 8A Score, 56.5 (9.2). **Postoperative results**: PROMIS Pain Intensity 1A Score, 4.1 (2.4), PROMIS Pain Intensity 3A Score, 49.7 (8.8), PROMIS Pain Interference 8A, 60.6 (8.2), PROMIS Emotional Distress-Anxiety 8A 52.3 (10.6)	PROMIS PI 1A Short form, PROMIS Pain Intensity 3A Short form, PROMIS PI 8A Short form, and PROMIS Emotional Distress-Anxiety 8A Short form	Level I
Bakhsh et al. [44]	Observational, retrospective cohort study	Unstable ankle fracture	209	NA	45 (range 16–84 years)	NA	PROMIS mood, under/uninsured 54, fully insured 49.1. PROMIS PF, under/ uninsured 38, fully insured 44.7. PROMIS PI, under/ uninsured 59.6, fully insured 55.5	PROMIS PF, PROMIS PI, PROMIS Mood	Level I
Bhashyam et al. [45]	Observational, retrospective study	Nonunion of the distal humerus after humerus fracture	7	42.9% (n = 3)	53.3 (range 41–75 years)	22 years (SD 3, range 19–27)	PROMIS PI 49.2 ± 9.1 (range 41.6–66.6), PROMIS Depression 49.7 ± 9.5 (range 41–63.6), PROMIS PF UE 41.8 ± 14.9 (range 19.3–58.2)	PROMIS PI, PROMIS Depression, PROMIS PF UE	NA
Bhashyam et al. [20]	Randomized controlled trial	Distal humerus fracture	76	67.1% (n = 51)	58 (range 22–94 years)	10.3 months (7.1)	PROMIS PF 41.7 ± 11.1, PROMIS PF UE 40.8 ± 12.4, PROMIS global (physical) 44.7 ± 11.6, PROMIS global (mental) 52.2 ± 10.4	PROMIS PF 10a, PROMIS PF UE 16a and PROMIS Global physical and Mental Health	Level I
Bozzio et al. [46]	Observational, retrospective cohort study	Acetabular fracture treated with closed reduction and percutaneous fixation	19	26.3% (n = 5)	47.5 (range 14–72 years)	18.7 months (range 15–29)	PROMIS PF mobility 66.4	PROMIS PF mobility	Level I
Carney et al. [47]	Observational, retrospective cohort study	Supination-adduction type II (AO/OTA 44A2.3) ankle fracture Observational, retrospective cohort study	65	46.1% (n = 30)	37 (14)	20.5 weeks (range 0.4–60.9)	PROMIS Physical Function 42.3 ± 11.3, PROMIS Pain Interference 55.8 ± 7.8	PROMIS PF and PROMIS PI	Level I, a private, academic tertiary referral center
Cavallero et al. [33]	Observational, retrospective cohort study	Bicondylar tibial plateau (BTP) fracture, complete articular, BTP fractures (AO/OTA 41- C and Schatzker 6)	46	47.8% (n = 22)	Locking group 51, Non-locking group 49	24.3 months (12–41)	Locking group, PROMIS PF 39, Non-locking group, PROMIS PF 41. Locking group, PROMIS PI 60, Non-locking group, PROMIS PI 57	PROMIS PF and PROMIS PI	Level I
Dean et al. [22]	Observational, retrospective cohort study	Closed ankle fracture	142	45.1% (n = 64)	52.7 (SD 14.7)	6.3 years (range 2–14)	PROMIS PF 51.9 (10.0), PROMIS PI 47.8 (8.45)	PROMIS PF and PROMIS PI	Level I
Eguia et al. [48]	Observational, retrospective cohort study	Supracondylar Humerus Fracture treated with lateral pinning or with crossed pinning	142	51% (n = 71)	5.2 (SD 2.0)	4.4 years (range 2–10)	Crossed pinning PROMIS PF UE 57 ± 6.2, PROMIS Pain interference 12 ± 0, PROMIS Strength impact 54 ± 1.6 Lateral Pinning (N = 93), PROMIS PF UE 56 ± 7.2, Pain interference 12 ± 3.2, Strength impact 53 ± 4.0	PROMIS Parent Proxy questionnaires: PROMIS PF UE CAT, PROMIS PI CAT and PROMIS Strength Impact short form A	Academic medical center
Eguia et al. [35]	Observational, retrospective cohort study	Supracondylar humerus fracture	213	49% (n = 104)	5.1 (SD 2.1)	5.0 (SD 2.1, range 2.0–10)	PROMIS PF UE 57 ± 5.5, 12 ± 2.1 for Pain Interference, PROMIS Strength Impact 54 ± 2.6	PROMIS Strength Impact, PROMIS PF UE and PROMIS PI	Tertiary care hospital
Evans et al. [49]	Observational, retrospective comparative study	Upper extremity fracture (wrist/hand, humerus, forearm, other)	297	32.3% (n = 96)	Least deprived quartile 12 (SD 2), most deprived quartile 12 (SD 3)	NA	NA	PROMIS Pediatric: PROMIS PF UE CAT, PROMIS PI CAT and PROMIS Peer Relationships CAT	Tertiary academic medical center
Fuchs et al. [50]	Observational, retrospective cohort study	Unstable ankle fracture	51	39.2 (n = 20)	Arthroscopy group 38.3, Non-arthroscopy group 40.3	67 months	PROMIS PF arthroscopy group 57.8, PROMIS PF non-arthroscopy group 54.5 PROMIS PI arthroscopy group 45.6, PROMIS PI non arthroscopy group 46.9	PROMIS PF CAT and PROMIS PI CAT	NA
Gausden et al. [21]	Observational, prospective cohort study	Unstable ankle fracture	132	59.8% (n = 79)	46.7 (SD 17.9)	NA	NA	PROMIS PF CAT and PROMIS LE PF CAT	NA
Gausden et al. [51]	Observational, prospective cohort study	Upper extremity fracture (olecranon, coronoid, radial head, and distal humeral fractures, humeral shaft, proximal humeral, or clavicular fracture)	174	58.6% (n = 102)	53.0 (range 15–90 years)	5.2 months (range, 1.3–16.7)	NA	PROMIS PF, PROMIS PI and PROMIS PF UE	NA
Gerull et al. [52]	Observational, cross-sectional study	Upper extremity fracture (humeral shaft, distal humerus, proximal forearm, distal forearm, unspecified, wrist/hand, forearm)	964	46.2% (n = 446)	NA	NA	PROMIS Upper extremity Parent proxy 30 (10, range 14–56, PROMIS Mobility Parent proxy Mean 45 (9, range 22–60), PROMIS Peer relationship Parent proxy 50 (10, range 15–66), PROMIS Pain interference Parent proxy Mean 54 (8, range 22–78). PROMIS PF UE self-administered 33 (11, range 14–57), PROMIS PF Mobility self-administered 44.9 (9, range 23–62), PROMIS Peer relationship self-administered 52.5 (10, range 17–66), PROMIS PI self-administered 48.7 (8, range 32–74)	PROMIS Parent Proxy questionnaires: PROMIS PF UE, PF Mobility, PROMIS PI and PROMIS Peer Relationships. PROMIS Pediatric: PROMIS PF UE, PF Mobility, PROMIS PI and PROMIS Peer Relationships	Tertiary orthopedic center
Gilley et al. [53]	Observational, prospective comparative study	Unstable ankle fracture	126	84.0% (n = 60)	45 (SD 14.0)	NA	PROMIS results mean (SD) [range]: isolated lateral malleolar (PF: 50 (11.4) [15.4–73.3]/PI: 51 (10.9) [38.7–83.8]), isolated medial malleolar (PF: 52 (8.2) [44.2–73.3]/ PI: 49 (8.4) [38.6–61.5]), bi-malleolar (PF: 47 (11.8) [26.3–73.3]/PI: 50 (11.5) [38.7–70.3]), tri-malleolar (PF: 48 (9.4) [24.1–73.3]/PI: 51 (8.3) [38.6–70.2]), isolated posterior malleolar (PF: 53 (7.7) [47.0–68.8]/PI: 44 (6.7) [38.7–56.0]), and isolated syndesmotic injury (PF: 60 (9.8) [49.8–73.3]/PI: 46 (7.5) [38.7–54.6])	PROMIS PF and PROMIS PI	NA
Glogovac et al. [54]	Observational, retrospective cohort study	Distal ulnar head and neck fracture	58	NA	56 (range 21–89 years)	27 months (range, 6–92 months)	distal ulna resection group PROMIS PF UE 34, non-operative group PROMIS PF UE 38, ORIF group PROMIS PF UE 45	PROMIS PF UE	Academic institution
Jayakumar et al. [36]	Observational, prospective longitudinal cohort study	Isolated distal radial fracture	364	78% (n = 284)	61 (SD 20, (range 18–99 years)	NA	**< 1 Week After Injury†:** PROMIS PF UE 21.2 (19.3–25.7), PROMIS PI 69.6 (63.1–73), PROMIS Depression 54.1 (46.1–58.6), PROMIS Anxiety 57 (44.3–58.1), PROMIS ES NM, PROMIS IS NM **2–4 Weeks After Injury†:** PROMIS UE 27.2 (23.9–30.9), PROMIS PI 65.6 (62.6–68.1), PROMIS Depression 46.1 (39.4–58.6), PROMIS Anxiety 42.7 (32.9–62.1), PROMIS ES 56.2 (39.6–58.8), PROMIS IS 57.6 (45.3 -59.2). **6–9 Months After Injury†:** PROMIS PF UE 43.9 (35–56.4), PROMIS PI: NM, PROMIS Depression NM, PROMIS Anxiety NM, PROMIS ES NM, PROMIS IS NM	PROMIS PF UE CAT, PROMIS Depression CAT, PROMIS Anxiety CAT, PROMIS PI CAT, PROMIS ES, PROMIS IS	Level I
Jayakumar et al. [55]	Observational, prospective longitudinal cohort study	Isolated proximal humerus, elbow, or distal radial fracture	744	66.9% (n = 498)	58.5 (SD 20.4, range, 18–97 years)	NA	**< 1 Wk. After Fracture**: PROMIS UE: 24.2 ± 6.2, Range 14.7–56.4, PROMIS PF: 32.4 ± 6.1, Range 23.5–55.8 **2–4 Wk. After Fracture:** PROMIS 29.2 ± 5.9, Range 15.6–45.7, PROMIS PF: 36.4 ± 8.4, Range 23.5–51.4 **6–9 Months After Fracture:** UE 43.2 ± 10.7, Range 19.5–56.4, PROMIS PF: 51.7 ± 12.8, Range 23.5–73.3	PROMIS PF and PROMIS PF UE	Level I
Jayakumar et al. [56]	Observational, prospective institutional review	Isolated proximal humerus, elbow, and distal radius fractures	744	66.9% (n = 498)	59 (SD 20)	NA	**PROMIS PF**: < 1 week after injury, 32 ± 6, At 2–4 weeks after injury 36 ± 8, At 6–9 months after injury 52 ± 13. **PROMIS UE PF**: < than 1 week after injury, number 744, Mean (SD) 24 ± 6, At 2–4 weeks after injury 29 ± 6, At 6–9 months after injury 43 ± 11	PROMIS PF and PROMIS PF UE	Level I
Jayakumar et al. [37]	Observational, prospective longitudinal cohort study	Isolated elbow fracture	183	49.7% (n = 91)	48.2 (SD 20.2; range, 18–93 years)	NA	PROMIS UE PF**: < 1 week after injury** 30.1 ± 6.4, Range 15.7–56.4, **Value at 2–4 weeks after injury** 34.3 ± 6, Range 21–45.7, **Value at 6–9 months after injury** 46.5 ± 10.1, Range 22.5–56.4 PROMIS PI: < **1 week after injury** 63.6 ± 7.3, Range (36.3–78.9), **Value at 2–4 weeks after injury** 57 ± 11.9, Range 0–71.6, **Value at 6–9 months after injury** 47.8 + 10.7, Range 34.8–74.1 PROMIS Depression: < **than 1 week after injury** 51.9 ± 13.2, Range (34.2–76.4), **Value at 2–4 weeks after injury** 47.6 ± 12.7, Range 34.2–76.3, **Value at 6–9 months after injury** 41.9 ± 10.8, Range 34.2–66.7 PROMIS Anxiety: < **1 week after injury** 49.1 ± 9.2, Range 32.9–69.1, **Value at 2–4 weeks after injury** 44.9 ± 10.8, Range 32.9–69.4, **Value at 6–9 months after injury** 40.8 ± 11.3, Range 32.7–67.1 PROMIS ES: < **1 week after injury** NM, Range NM. **Value at 2–4 weeks after injury** 54.7 ± 9.3, Range 31.5–66.9, **Value at 6–9 months after injury** 54.2 ± 9, Range 33.6–66.2 PROMIS IS: < **1 week after injury:** NM, Range NM, **Value at 2–4 weeks after injury** 54.8 + 7.4, Range 35.1–66.1, **Value at 6–9 months after injury** 54.7 ± 6.6, Range 41.2–62.1	PROMIS PF UE, PROMIS PI, PROMIS Depression, PROMIS Anxiety, PROMIS ES and PROMIS IS	Level I
Jayakumar et al. [38]	Observational, prospective cohort study	Isolated fracture of the proximal humerus, elbow, or distal radius	744	66.9% (n = 798)	58.5 (SD 20.4; 18–97 years)	NA	NA	PROMIS PI, PROMIS Depression, PROMIS Anxiety, PROMIS ES and PROMIS IS	Level I
Jayakumar et al. [39]	Observational, prospective longitudinal cohort study	Proximal humeral fracture	177	72.3% (n = 128)	66 (SD 16; 18- 95 years)	NA	PROMIS UE PF CAT: **Value at < 1 wk. after injury**, 21.9 (5.5; 14.7–40.7), **Value at 2–4 wks. after injury**, 27.1 (5.4; 15.6–41), **Value at 6–9 mths after injury**, 40.5 (9.9; 26.2–56.4). PROMIS PI: **Value at < 1 wk. after injury**, 68 (6.8; 38.1–76.9), **Value at 2–4 wks. after injury**, 66.9 (5.9; 46.1–74.1), **Value at 6–9 mths after injur**y, 52.8 (11.2; 38.7–70.3). PROMIS Depression: **Value at < 1 wk. after injury**, 50.7 (9.5; 34.2–75.6), **Value at 2–4 wks. after injury**, 50.2 (9.6; 34.2–69.2), **Value at 6–9 mths after injury,** 45.1 (11.5; 34.2–68). PROMIS Anxiety: **Value at < 1 wk. after injury**, 52.2 (11.6; 32.9–76.2), **Value at 2–4 wks. after injury**, 47 (10.4; 32.9–69.6), **Value at 6–9 mths after injury**, 44.2 (11.2; 32.9–63.3). PROMIS ES: **Value at < 1 wk. after injury,** NM, **Value at 2–4 wks. after injury**, 51.6 (12.1; 32.6–66.2), V**alue at 6–9 mths after injury**, 51.3 (11.4; 31.5–66.2). PROMIS IS: **Value at < 1 wk. after injury**, NM, **Value at 2–4 wks. after injury**, 55 (9.5; 38.8–66.2), **Value at 6–9 mths after injury**, 53.5 (9.1; 38.8–66.2	PROMIS PF UE, PROMIS PI, PROMIS Depression, PROMIS Anxiety, PROMIS ES and PROMIS IS	Level I
Kaat et al. [57]	Observational, prospective cross-sectional and longitudinal study	Complete intra-articular distal humeral fracture	424	53,9% (n = 228)	47,3 (SD 17,4)	NA	**PROMIS UE-CAT**, overall, Time 1: 32.8 (SD 9,5), Time 2: 42.5 (SD 8.6). **PROMIS PF-SF8a**, Overall, Time 1: 41.6 (SD 6.9), Time 2: N 131, 48.9 (SD 7.5)	PROMIS PF UE CAT and PROMIS PF short form 8a	Level I
Kaiser et al. [58]	Observational, retrospective cohort study	Isolated distal radial fracture	56	71.4% (n = 40)	ORIF with olecranon osteotomy 76.9 (range 65–92). Limited fixation 79.8 (range 65–96)	15.2 months (range 12–97 months)	**PROMIS PI**: ORIF with osteotomy, 53.1. Limited fixation group 52.14. **PROMIS PF**: ORIF with osteotomy, 41.7. Limited fixation group 41.1	PROMIS PF and PROMIS PI	Orthopedic trauma center
Kempton et al. [59]	Observational, prospective cohort study	Surgically treated tibia plateau fracture	183	na	Group 1: 50.0, Group 2: 50.3	group 1: 31 months, group 2: 15 months	**PROMIS PF**: Group 1, 41.2. Group 2, 42.8 **PROMIS PI:** Group 1, 55.9. Group 2, 55.6	PROMIS PF and PROMIS PI	Level I
Kohring et al. [60]	Observational, prospective cohort study	Ankle fracture ORIF and syndesmotic fixation	71	na	SSR group at initial ORIF: 43 (SD 17). cohort comparison group: 44 (SD 18)	106 days (SD 44)	**PROMIS PF:** after ORIF before SSR 35.2 (SD 8.0, range 19.3–61.7). **PROMIS PI**: pre-screw removal 56.5 (SD 9.6, range 32.2–73.7). **PROMIS depression**: 46.2 (SD 9.6, range 31.9–65.7). **PROMIS PF**: post-screw removal 44.5 (SD 7.7, range 26.9–61.7). **PROMIS PI**: post-screw removal 54.1 (SD 9.2, range 32.2–71.6). **PROMIS depression**: 43.4 (SD 10.1, range 31.9–67.8)	PROMIS PF, PROMIS PI and PROMIS Depression	Level I
Metcalf et al. [61]	Observational, retrospective cohort study	Intra-articular distal tibia fracture	135	45% (n = 60)	45.3 (range 16–84)	9 months in the extra-articular group and 10 months in the intra-articular group	**PROMIS PI:** extra articular group 55.1 (SD 7.8). Intra-articular group 59.4 (SD 9.3) **PROMIS PF**: extra articular group41.9 (SD 7.6), intra-articular group 42.3 (SD 8.4)	PROMIS PF and PROMIS PI	Level I
Minoughan et al. [62]	Observational, prospective study	fracture of the shoulder n = 7, Adhesive capsulitis (n = 13), Failed arthroplasty (n = 7), Instability (n = 23), Impingement syndrome (n = 5), Rotator cuff disease (n = 31), Other (n = 4)	90	45.6% (n = 41)	50.3 (SD 17.3, range 14–90)	NA	PROMIS PF UE: 34.9 (SD 9.6, range 14.7–61)	PROMIS PF UE	Level I
Morgan et al. [63]	Observational, prospective study	Proximal humeral fractures	47	61.7% (n = 29)	68.0 (range 60–88)	NA	PROMIS PF CAT 44.4	PROMIS PF CAT	Level I
North et al. [64]	Case series	Ankle fracture	3	66.7% (n = 2)	39 (range 22–48)	NA	PROMIS PF scores at **2 wk. appointment:** pt. 1: 33.27, pt. 2: 72.82, pt. 3: 24.67. PROMIS PF at **6 wk. appointment**: pt. 1: 41.35, pt. 2: 37.91, pt. 3 34.93. PROMIS PF **at 12 week appointment**: pt. 1: 38, pt. 2: 40.27, pt. 3: 43.0	PROMIS PF CAT	Level I
Ochen et al. [65]	Observational, retrospective cohort study	AO/OTA 41-C or Schatzker V/ VI tibial plateau fractures	216	49.5% (n = 107)	53 (SD 13, range 24–89)	86 months from injury (IQR; 48–134)^a^	PROMIS PF: 47.7 (SD 9.5)	The PROMIS PF short-form-10	Level I
Okike et al. [66]	Observational, retrospective cohort study	Proximal humeral fractures	207	25.1% (n = 52)	76.9	3.3 years	PROMIS PF CAT: non operative group 43.9, operative group 45.0	PROMIS PF CAT	NA
Okoroafor et al. [67]	Observational, cross-sectional evaluation	Upper extremity fractures (humeral shaft, distal humerus, proximal forearm, distal forearm, unspecified forearm, and wrist/hand)	975	NA	Quartile 1 Least Deprived: 12.2 (SD 2), Quartile 2: 12.1 (SD 3), Quartile 3: 12 (SD 3), Quartile 4 Most Deprived: 11.7 (SD 2)	NA	**PROMIS PF UE**: Quartile 1 Least Deprived: 39 (11). Quartile 2: 37 (11), Quartile 3: 36 (10), Quartile 4 Most Deprived: 35 (11). **PROMIS PF Mobility CAT:** Quartile 1 Least Deprived: 48 (10) Quartile 2: 47 (9) Quartile 3: 46 (9) Quartile 4 Most Deprived: 44 (9) **PROMIS PI CAT:** Quartile 1 Least Deprived: 46 (8), Quartile 2: 46 (8) Quartile 3: 48 (8), Quartile 4 Most Deprived: 50 (8) **PROMIS Peer Relation CAT:** Quartile 1 Least Deprived: 55 (10) Quartile 2: 53 (10) Quartile 3: 52 (9) Quartile 4 Most Deprived: 50 (9)	PROMIS Pediatric: PROMIS PF UE CAT, PROMIS PF Mobility CAT, PROMIS PI CAT and PROMIS Peer Relation CAT	Tertiary orthopedic center
Ozkan et al. [68]	Double-blind, placebo-controlled randomized trial	Distal radius fracture	134	73.9% (n = 99)	49 (SD 17)	6.46 months (SD 0.91 months, range, 5–9 months)	**PROMIS PI:** total cohort 63 (SD 7.0), Vitamin C group 65 (SD 6.4) placebo group 61 (SD 7.1) **PROMIS PF UE:** total cohort 27 (SD 7.7), vitamin C group 26 (SD 7.9), Placebo group 27 (SD 7.5)	PROMIS PF UE and PROMIS PI	Level I
Pet et al. [40]	Observational, retrospective study	Proximal pole scaphoid nonunion	41	14.6% (n = 6)	24.1 (SD 5.4, range, 16–40 years)	2.9 years (SD 1.8)	PROMIS PF UE: 50.1 PROMIS PF: 54.7 PROMIS Global Health: 56.1 PROMIS Pain intensity: 37.1 PROMIS PI: 46.5 PROMIS Pain behavior: 44.3	PROMIS PF UE, PROMIS PF, PROMIS Global and mental Health, PROMIS Pain Intensity, PROMIS PI and PROMIS Pain Behavior	NA
Rothrock et al. [34]	Observational, prospective cohort study	Isolated lower extremity fracture (ankle/foot, tibia/fibula, patella, femur, pelvis)	402	44.0% (n = 177)	Time 1: 45, 1, (SD 16.9). Time 2: 46.7, (SD 15.2)	80 days (range 12–364 days)^a^	PROMIS PF Mobility CAT: 35.5 (SD 8.5), PROMIS PF SF8a 34.2 (SD 9.1)	PROMIS PF Mobility CAT and PROMIS PF short Form 8a	Level I
Sandvall et al. [69]	Observational, retrospective cohort study	Distal radius fracture	187	82.9% (n = 155)	56 (SD, 20; range, 18–94 years)	35 days (IQR, 25–45)^a^	PROMIS Physical Function 37 (SD 10)	PROMIS PF CAT	NA
Shah et al. [70]	Observational, retrospective cohort study	High-energy and low-energy injuries	333	62.2% (n = 207)	High Energy trauma 68.78 (SD 6.83), Low Energy trauma 76.17 (SD 9.29)	NA	PROMIS PF, High-Energy Injury: 42.16 (SD 10.41), Low-Energy Injury: 24.64 (SD 10.45)	PROMIS PF	Level I
Sharma et al. [41]	Observational, retrospective cohort study	Extra-articular scapula fracture (scapula, clavicle, and/or glenoid fracture)	5	0% (n = 0)	Scapula fracture group 65.4. control group 62.4	5.6 years (range 3.2–9.2 years)	PROMIS global health PF for patients 50.0, control group 57.7 PROMIS global health mental for patients 50.8, control group 67.6 PROMIS PF SF12a for patients 52.4, control group 52.4	PROMIS PF short form 12a and PROMIS Global Health PF and Mental	NA
Smith et al. [42]	Observational, retrospective cohort study	Ankle fracture	213	54.5% (n = 116)	ORIF with Arthroscopy: 39.9, ORIF Alone: 40	32.4 months	PROMIS global health physical function ORIF group: 42.7, ORIF + arthroscopy group 44.9 PROMIS global health mental ORIF group: 46.2, ORIF + arthroscopy group 47.1	PROMIS Global Health Short Form and PROMIS PF	NA
Stuart et al. [71]	Observational, prospective cohort study	Heterogeneous fracture types (Acetabulum, bi-malleolar ankle fracture, clavicle, distal humerus, distal radius, femoral neck, femoral shaft, fibula,intra-articular elbow, medial malleolus, metatarsal, patella, pelvis, posterior malleolus, proximal humerus, sub trochanteric femur, talus, tibia shaft, tibia plafond, tibia plateau, tri-malleolar ankle fracture and ulna,	50	32% (n = 16)	Patient age: 42.7 (SD 16.1; range 18–71 years). Proxy age 49.8 (SD 12.8; range, 20–78 years)	14.3 days (SD 1.06; range 14–18 days)	Patient’ perceived preinjury PROMIS PF CAT 57.9 (SD 10.4). Proxies’ perceived preinjury PF CAT 56.6 (SD 11.5)	PROMIS PF CAT	NA
Swarup et al. [72]	Observational, retrospective cohort study	Posterior sternoclavicular physeal fractures and dislocations	37	10.8% (n = 4)	15.2 (SD 2.1, range 5.8–17.7 years)	4.5 years (SD 3.4, range: 1.0–10.6 years)	PROMIS PF UE: 55 (SD 3.5, range: 48 -57)	PROMIS PF UE	Tertiary referral center for pediatric trauma
Vd Vliet et al. [73]	Observational, retrospective cohort study	Subtalar arthrodesis for posttraumatic arthritis after a calcaneal fracture	159	37.1% (n = 59)	48 years (IQR, 39–55 years)^a^	8.8 years (IQR, 4.3–12.2 years; range, 1.1–15.6 years)^a^	PROMIS PF: 45, (IQR 38–51)^a^	PROMIS PF Short Form 10a	Level I
vd Vliet et al. [74]	Observational, retrospective cohort study	Open reduction internal fixation for tibial pilon fracture	225	39% (n = 88)	48 (IQR, 37–58 years)^a^	82 (IQR, 45–120 months)^a^	PROMIS PF: 49 (IQR 44–57)^a^	PROMIS PF Short Form 10a	Level I
v Leeuwen et al. [75]	Observational, prospective cohort study	Orthopedic trauma patients (fractures)	124	50% (n = 62)	54 (SD 19, range: 18- 93 years)	NA	PROMIS PF: 36 (9.5), 95% Confidence interval 34–38 PROMIS PI: 50 (8.4), 95% Confidence interval 48–51	PROMIS PF CAT and PROMIS Pain Intensity	Tertiary care hospital
van Wyngaarden et al. [76]	Observational, prospective cohort study	Lower extremity fracture (pelvis/acetabulum, femur, tibia, patella, ankle/foot)	122	45% (n = 52)	42.1 (SD, 14.6)	NA	PROMIS Depression: 54.2 (SD 9.1) PROMIS PI 59.1 (SD 7.7)	PROMIS Depression CAT and PROMIS PI CAT	Level I
Verhiel et al. [77]	Case series	Essex-Lopresti injury (ELI)	16	12.5% (n = 2)	42 (SD 10)	10 years (IQR, 8.0–12) years)^a^	PROMIS PF UE: 36 (IQR: 33–38). Conservative treatment PROMIS PF UE 41 (IQR: 32–50), operative treatment PROMIS PF UE 33 (IQR: 33–37)^a^	PROMIS PF UE	Level I and community hospital
Vincent et al. [78]	Randomized controlled study, secondary observational analysis	Lower body fractures (tibia/fibula, femur, pelvis, patella, metatarsals) and upper extremity fractures (radius, ulna, humerus)	101	40.6 (n = 41)	43.5 (SD 16.4, range 40.2–46.7 years)	NA	PROMIS PF: **acute care** total cohort 27.0 (SD 7.1), No Depression 26.3 (SD 7.4), Depression 29.4 (SD 6.7). **2 weeks** total cohort 31.6 (SD 6.2), No Depression 31.6 (SD 6.2), Depression 31.6 (SD 6.2). **6 weeks** total cohort 33.2 (SD 6.2), No Depression 33.4 (SD 6.5), Depression 32.7 (SD 4.6). **12 weeks** All 37.8 (SD 7.1), No Depression 38.3 (SD 7.3), Depression 35.3 (SD 5.2) PROMIS satisfaction with social roles and activities: **acute care** total cohort 40.7 (SD 10.1), No Depression (SD 10.0), Depression 37.5 (SD 10.3). **2 weeks** total cohort 43.9 (SD 8.11), No Depression 44.4 (SD 8.5), Depression 42.1 (SD 5.6). **6 weeks** total cohort 45.9 (SD 8.4), No Depression 47.1 (SD 7.9), Depression 40.3 (SD 8.8). **12 weeks** total cohort 50.0 (SD 10.2), No Depression 50.8 (SD 10.0), Depression 45.6 (SD 10.1) PROMIS Psychosocial illness impact-positive, **Acute care** total cohort 52.8 (SD 9.9), No Depression 54.6 (SD 9.6), Depression 44.2 (SD 6.5), **2 weeks** total cohort 53.6 (SD 9.5), No Depression 54.8 (SD 9.8), Depression 47.8 (SD 5.1), **6 weeks** total cohort 54.6 (SD 10.7), No Depression 56.1 (SD 10.3), Depression 47.1 (SD 10.0), **12 weeks** total cohort 55.8 (SD 12.7), No Depression 58.1 (SD 8.8), Depression 44.8 (SD 12.7)	PROMIS PF, PROMIS satisfaction with social roles and activities and PROMIS psychosocial illness impact-positive	Level I
Virkus et al. [79]	Observational, retrospective cohort study	OTA/AO 41-C (Schatzker 6) BTP (Bicondylar tibial plateau) fracture treated with open reduction and internal fixation	52	40.4% (n = 21)	One-Stage Fixation 48 years, Two-Stage Fixation 51 years	21.8 months (range 6–41 months)	PROMIS PF: One-Stage Fixation 40, Two- Stage Fixation 40. PROMIS PI: One-Stage Fixation 61, wo- Stage Fixation 56	PROMIS PF and PROMIS PI	Level I
Wilkens et al. [80]	Observational, cross-sectional study	Finger, hand, or wrist injury (finger fracture, metacarpal fracture, wrist fracture, finger sprain finger, laceration, mallet fracture, carpal bone fracture, or wrist sprain.)	149	51% (n = 76)	46 (IQR 28–61 years)^a^	NA	PROMIS PF UE: Hand posture Yes 32 (SD 8), Hand posture NO 34 (SD8) PROMIS Depression CAT: Hand posture Yes 48 (IQR 41–55), Hand posture NO 48 [IQR 42–530 PROMIS PI: Hand posture Yes 59 (IQR 56–64), Hand posture NO: 59 (IQR 54–63)^a^	PROMIS PF CAT, PROMIS PI CAT, PROMIS PF UE and PROMIS Depression	Tertiary care hospital

*PI* pain interference, *PF* physical function, *PF UE* physical function upper extremity, *UE* upper extremity, *CAT* computer adapted testing, *NA* not applicable, *LE PF* lower extremity physical function, *ES* emotional support, *IS* instrumental support, *NM* not mentioned, *SSR* syndesmotic screw removal, *ORIF* open reduction and internal fixation, T-scores: range from 0 to 100, mean of 50, SD of 10 points

A higher score for positive constructs is better (i.e. PF, PF UE, PF mobility, LE PF, ES, IS, global and mental health, psychosocial illness impact-positive, satisfaction with social roles and activities, strength impact, peer relationships and mood) and worse for negative constructs (i.e. PI, Pain Intensity, pain behaviour, depression and emotional distress-anxiety)

^a^Median follow-up/age/PROMIS score. T-scores: range from 0 to 100, mean of 50, SD of 10 points

**Fig. 2:**
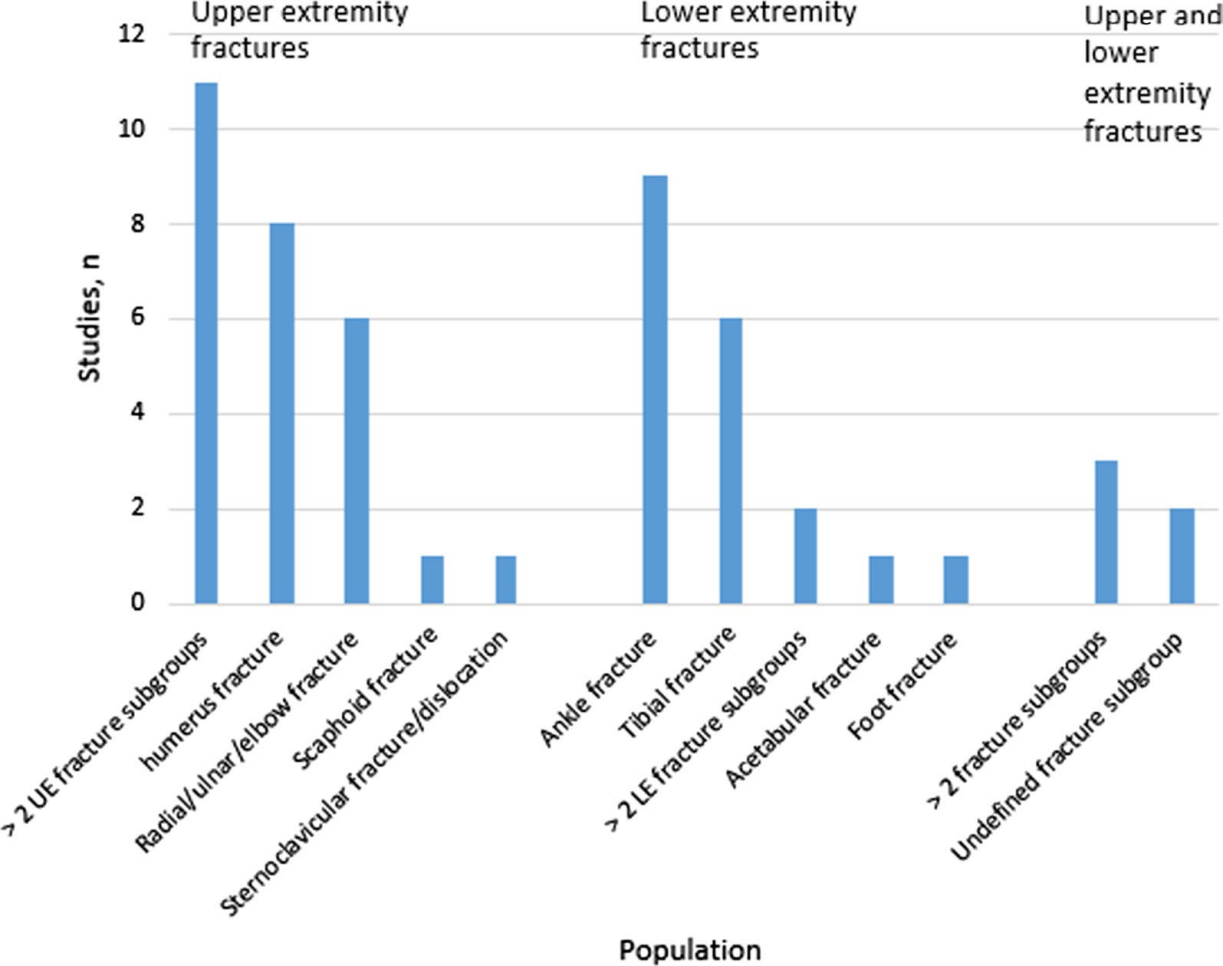
Population and fracture subtypes included in this paper. UE: upper extremity, LE: lower extremity

A single fracture population was included in 47 studies of which 9 involved ankle fractures (9/51; 17.6%), followed by humeral fractures (8/51; 15.7%), tibia fractures (6/51; 11.8%) and radial or ulnar fractures (5/51; 9.8%). Less than a third of all papers involved more than 2 fracture subgroups (16/51; 31.4%) and 3 papers (3/51; 5.9%) did not specify their subgroup (Fig. 2). Sample sizes ranged from 7 to 975 and most studies only included patients ≥ 18 years old in their papers (39/51; 76.5%). The patients’ age ranged from 1 [35] to 99 [36] years.

**Fig.3:**
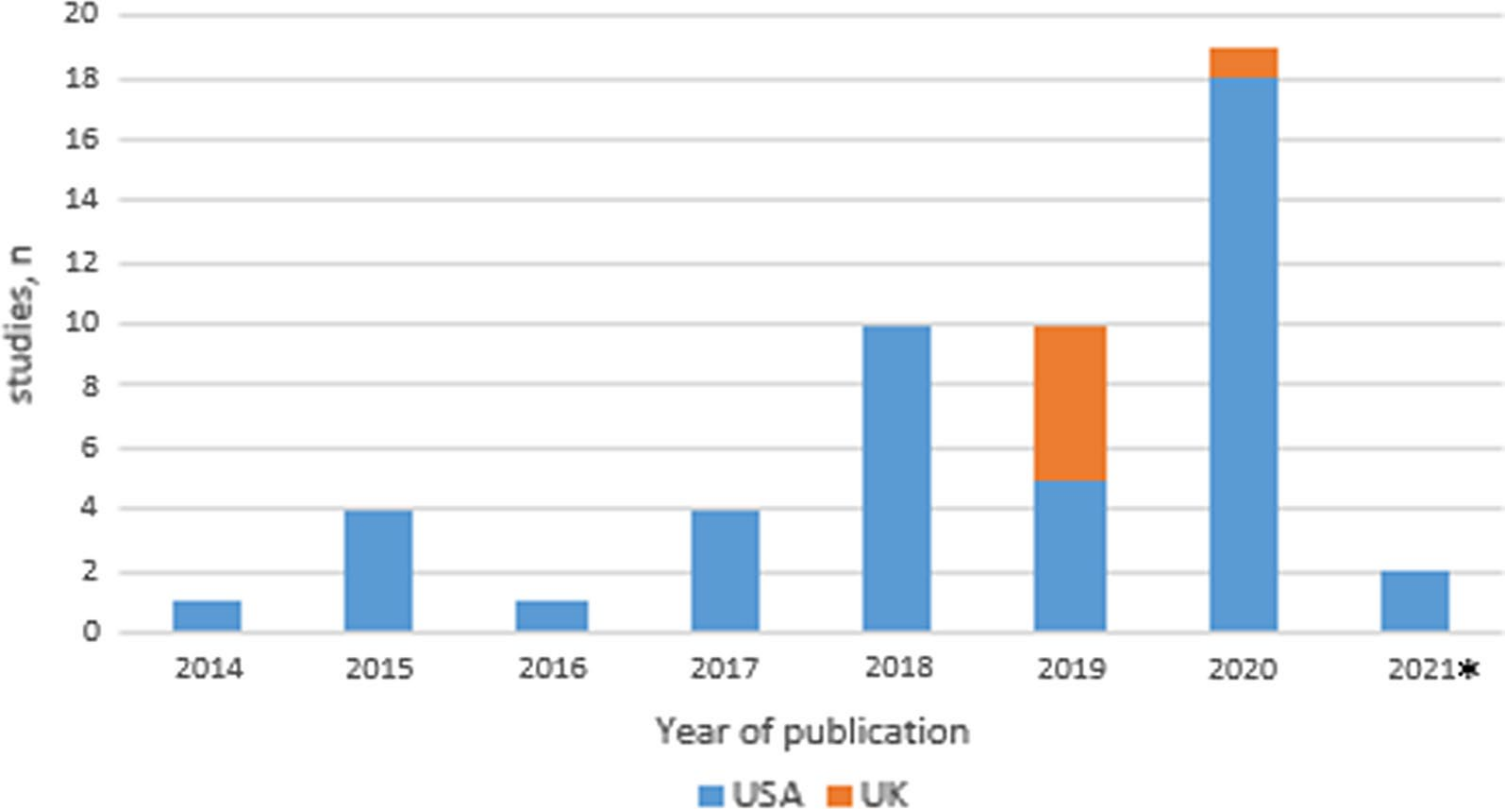
The number of full text papers published about a fracture subpopulation divided by country. *The included publications from the year 2021 were pre-view articles which were published in January 2021

Included articles in this review showed a gradually increasing frequency of PROMIS questionnaires in the orthopedic fracture population since 2017, with already 2 published papers in January 2021. All studies were conducted in the USA (46/51; 88.2%) or UK (6/51; 11.8%) (Fig. 3). Most studies were performed in a level 1 -or tertiary care trauma center (37/51; 72.5%) (Table 1).

### PROMIS measures

**Fig.4:**
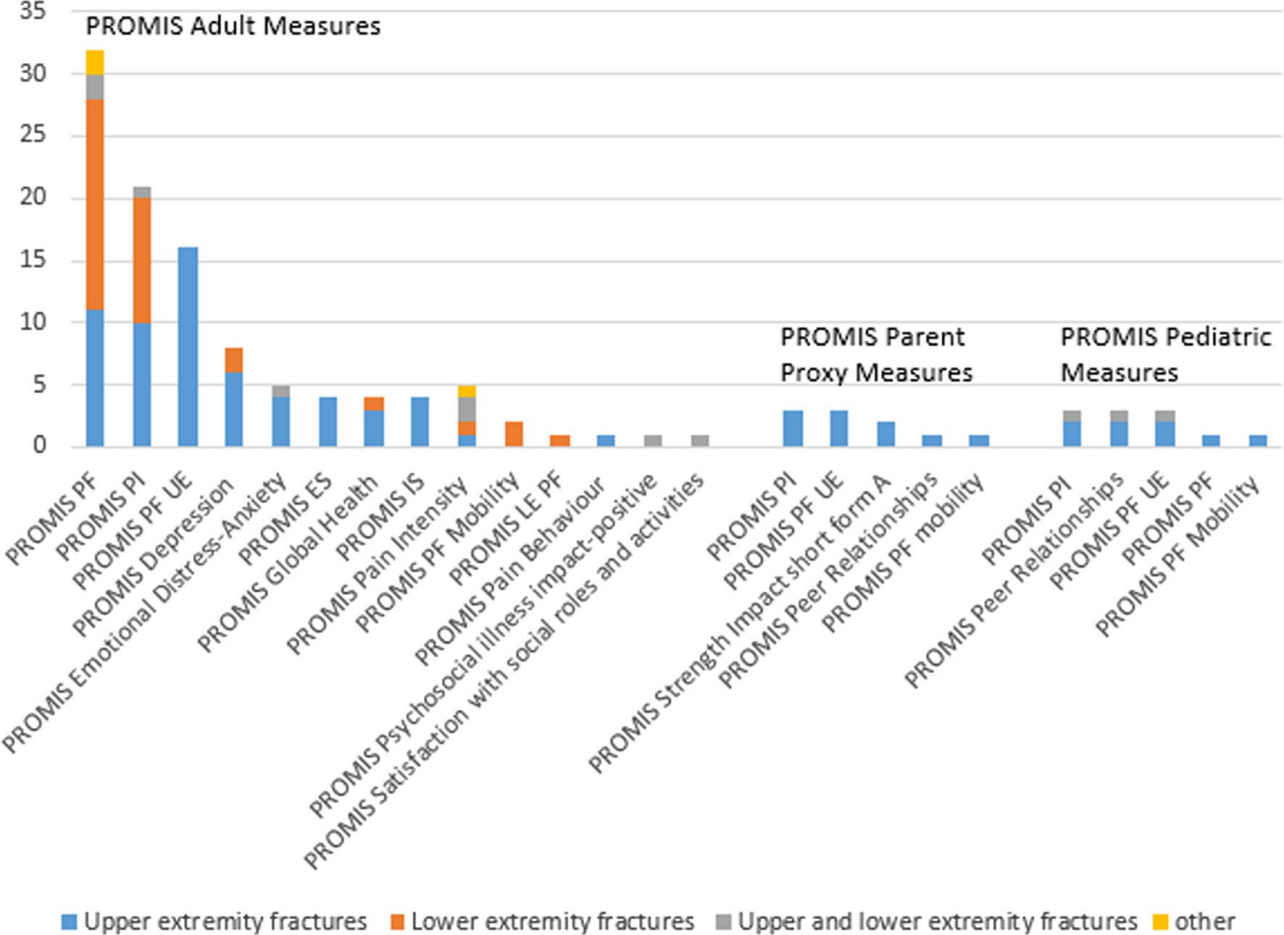
PROMIS measures divided by subgroups for adult PROMIS measures, PROMIS parent proxy measures and pediatric PROMIS measures. PF: Physical Function, PI: Pain Interference, PF UE: Physical Function Upper Extremity, ES: Emotional Support, IS: Instrumental Support, LE PF: Lower Extremity Physical Function, other: not specified if upper or lower extremity fractures

Figure 4 shows all used PROMIS measures divided by subgroups for the adult PROMIS measures, PROMIS parent proxy measures and pediatric PROMIS measures. For both the upper and lower extremity fractures, the PROMIS Physical Function (n = 32, 32/51 = 62.7%) and PROMIS Pain Interference (n = 21, 21/51 = 41.2%) were most frequently used questionnaires in the physical health domain. PROMIS Physical Function Upper Extremity (n = 16, 16/51 = 31.4%) was most frequently used in the upper extremity group. PROMIS measures concerning social and mental health were much less often used as outcome measures in the fracture population. In the mental health domain, one research group accounted for more than one third (n = 4, 4/10 = 40%) of all mental health questionnaires in multiple papers [36–39], i.e. PROMIS depression, PROMIS emotional distress- anxiety and PROMIS psychosocial impact-positive. The upper extremity population was most frequently asked about mental health. The social health domain was studied in 5 different papers and again, 4 papers were published by the same research group [36–39]. The studied social health domain included: emotional support, instrumental support and satisfaction with social roles and activities. As an overall evaluation of a patients’ physical and mental health, the PROMIS global health questionnaire was studied in 4 articles [20, 40–42].

Additional papers concerning the pediatric population and pediatric measures, primarily focused on PROMIS® pain interference, PROMIS physical function upper extremity and PROMIS peer relationships. On top of this PROMIS physical function and PROMIS physical function mobility were used as outcome measures.

## Discussion

The primary goal of this systematic literature review was to provide an overview of PROMIS measures in the orthopedic fracture population. The aim was to determine the frequency and extensiveness of usage of available PROMIS measures (physical health, mental health and social health). Secondly, we assessed the use of PROMIS differentiated by type of fracture to evaluate if PROMIS is more often used in specific fracture types.

The systematic review shows that some fracture populations were in particular subject of research in the use of PROMIS measures. Fractures of the ankle, humerus, tibia and radius/ulna and elbow account for more than 50% of all included papers. The popularity of these fracture populations could possibly be found in the high incidence of specific fractures and the previous use of legacy measures/fixed scales other than PROMIS measures for these populations [7, 81] in combination with the validation of PROMIS measures for these certain groups [82].

Sex differentiation in this review showed a female proportion of 0–78% in included studies. Twenty included studies showed a female proportion of ≥ 50% and ten studies showed a female proportion of ≥ 45%. As fractures show a gender -and age specific pattern in which males are more often exposed to fractures in the age from 12 to 50 and females exceed the males from 50 years of age, it could generally be noticed that females were adequately represented [83]. Spread in follow up time was large and documentation of PROMIS measures diverse. Ideally, the use of PROMIS measures or PROMs in general could generate data on regular dates to enlarge generalizability and comparison between data. Only research performed in level I trauma centers or academic/tertiary centers were seen in this systematic review. For generalizability of the fracture population, it would be better to also include patients from non-trauma or non-academic centers.

The results also show that the physical health domain was most often used in the assessment of orthopedic fracture patients. Especially physical function and pain interference were highlighted in the included papers. A recent systematic review showed that PROMIS physical function strongly correlated with other frequently used orthopedic PROMs in upper -and lower extremity patients [23]. Orthopedic fracture patients are in the current healthcare system being assessed in the emergency department in the acute setting, in (virtual) fracture clinics for follow up after the emergency department and in consultant specialty fracture clinics in the case of more complex fractures for follow-up or additional (surgical) treatment [84]. Since physical function and pain is the main measure of progress for orthopedic conditions, it is comprehensible to firstly evaluate this domain. Patients seen in the acute phase could primarily be provided with PROMIS questionnaires physical function and pain, but for follow up in fracture clinic or specialty consultant fracture clinic, additional questionnaires regarding social and mental aspects could be of value to patient centered care.

As seen in the results of our study, social health and mental health were assessed in the minority of included articles. Traditionally, outcomes in trauma patients focused on in-hospital parameters and survival [85]. As survival to discharge improved, post discharge and long term quality of life outcomes became more important to injured patients [14, 15]. Yet, as this systematic review shows, papers merely focus on the physical health domains. Increasing evidence is found that health aspects other than physical functioning are important for recovery to patients with one or more bone fractures [86]. Firstly, mental health could be limited by depressive or anxious symptoms after trauma, which are noted for reducing the overall quality of life, but mental health also tends to limit physical progression and extend the usage of pain medication [16, 17]**.** Secondly, patients with bone fractures have to deal with social insecurities. Patients could be uncertain about the future, the ability to work, the need for social support and their own view on their body image [87, 88]. Early intervention into social and mental health problems is known for reducing the number of hospital readmissions [89], improving reintegration into the community [90] and improving overall health related quality of life after a trauma [91]. So, patients seen in specialty fracture clinics with more complex fractures, slow progress in physical recovery or complications are prone to have additional health problems and needs and could therefore be supported with additional PROMIS measures exploring social and mental health.

PROMIS measures, including social and mental health, can easily be explored by short forms and CAT-versions in which the response burden for participants and clinicians is limited and estimates be more precise [92]. Clinicians are able to be informed in advance of outpatient clinic visits on a patients’ health status and can anticipate on possible problems. Simultaneously, implementation of PROMIS measures as part of the electronic medical report (EMR) to summarize health status of the patient is possible. This potentially limits administrative tasks for physicians. On top of this, progression on different health domains can be visualized by implementation into the EMR which improves understanding and discussion with the patient [93]. By showing results to patients, the threshold to discuss difficulties in physical, mental and social health could be lowered and patients could feel supported as already seen in studies with multi-morbidity patients [94]. The orthopedic trauma surgeon could also use health information to refer patients to supporting departments, such as the pain management specialist, the psychology department or social work, and in return, these departments can read and interpret the outcomes of PROMIS measures too. Simultaneously, comparison between PROMIS measures in fracture subgroups is easier, since PROMIS scores are generalizable, efficient and highly reliable. Lastly, the disadvantage of low generalizability in several different legacy PROMs to assess injuries in similar anatomical locations can be tackled by the use of PROMIS [4, 7, 23].

Results of our study showed a preference for research of mental health constructs in patients with upper extremity fractures. No conclusive explanation could be found to this observation, since both upper extremity injuries as lower extremity injuries are associated with limitations in mental health (and social health and physical health) [95–98]. Future research could therefore focus on differences between mental health outcomes in upper extremity fracture patients and lower extremity fracture patients by use of PROMIS measures. Furthermore, future research could also focus on the impact of trauma on social aspects of life (e.g. work, ability to participate in the community, support preferences, social interaction after trauma and reaching the preliminary social status). On top of this, the effect of the use of PROMIS in outpatient fracture clinics on the felling of support and health related quality of life could be expanded.

Included articles were conducted in the United States and the United Kingdom. Papers from other countries were available in the primary/initial search, but did not meet our inclusion criteria. For example because of inclusion of heterogeneous populations not specified to fracture patients. Nevertheless, articles from English speaking countries with PROMIS-networks like Canada and Australia were lacking. In Canada, PROMIS measures are translated into French as well, so this could potentially result in less hits with regard to PROMIS and fracture patients, but no French articles were found in our search. In Australian literature, especially the use of PROMIS-29 and PROMIS 10 in for example the New South Wales Trauma Outcome Registry and Quality Evaluation (TORQUE) was promoted, but as far as our search reaches, no publications were generated from these reports [99, 100]. PROMIS-networks in other non-English speaking countries have also been established in for example the Netherlands and Belgium (Dutch-Flemish PROMIS group), and Germany (PROMIS® Germany). These PROMIS communities provide information about PROMIS, support development and translation of PROMIS measures and can advise or participate in new scientific projects. Yet, it has to be emphasized that the development and translation of PROMIS measures to languages other than English, needs to be continued for worldwide implementation. Especially the measurement and documentation of PROMS in low and -middle income countries (LMIC) in general, is running behind [101, 102]. In our case, articles including PROMIS measures and fracture patients from LMIC are absent, but the PROMIS Health Organization does show that PROMIS translations (in fixed length or CAT version) are available to every continent of the world [103]. Barriers for the successful introduction and usage of PROMIS could possibly be found in more sophisticated technologies in the use of computer adaptive testing which may not be routinely available, but barriers of PROs in general could be linked to missing centralized documentation in EMR or financial support [101]. We would therefore advocate to both scientists and clinicians to explore the possibilities of PROMIS within their own PROMIS communities for the prospects of PROMIS.

This systematic review has a few limitations. Firstly, articles were possibly missed during database search. But, by involving a professional librarian and by searching in five medical databases, we attempted to include all available articles. Furthermore, only English papers were included which theoretically increases the risk for language bias. Language bias is actually very limited, since only two papers were excluded due to the non-English language. Thirdly, only one reviewer independently extracted the data which could potentially cause information bias, but a second reviewer checked randomly for accuracy and supplemented if needed. Fourthly, the STROBE-checklist was used for the assessment of observational studies. However, originally it has been developed as a checklist for the report of observational studies, rather than a tool for methodological quality assessment [30]**.** No universal consensus exists in the assessment of methodological quality of observational studies, but the use of the STOBE checklist does support in the knowledge and evaluation of observational papers [104, 105]. Furthermore we faced difficulties in the assessment of papers by use of the strobe checklist or by use of the care guidelines for case series. Multiple papers mentioned the term ‘case series’ in their abstract, but classified the paper in the main text as cohort study. As known from the literature, distinguishing cohort studies and case series might be difficult [106]. Therefore, we only chose to assess papers by using the care guidelines for case series if the title or main text involved a description with the term case series. Lastly, readers should take into consideration that the upward trend of the use of PROMIS measures will probably increase, because results as shown in this paper apply until January 2021.

## Conclusion

This review shows that the use of PROMIS measures in the field of orthopedic fracture care is increasing. Many different PROMIS measures on multiple domains are available and already used in previous articles for the evaluation of patient outcomes. With physical function and pain interference as most popular PROMIS measures, it is important to emphasize that other health-domains such as mental and social health can also be essential to fracture patients. PROMIS measures offer a valid, reliable and easy accessible tool to evaluate the patient as a whole and healthcare providers providing care for fracture patients (and scientists) could consider to connect to available national PROMIS-networks to learn more about the possibilities of PROMIS.

## Supplementary Information

**Additional file 1.** Full search strategy.

**Additional file 2.** STROBE checklist.

**Additional file 3.** Care guidelines.

**Additional file 4.** Downs and Black Checklist.

## Data Availability

Data are available from the authors upon reasonable request.
