# Supplementary material for: There are more things in physical function and pain: a systematic review on physical, mental and social health within the orthopedic fracture population using PROMIS
Source: J Patient Rep Outcomes. 2022 Apr 6;6:34. doi: 10.1186/s41687-022-00440-3 (PMC8986932; doi:10.1186/s41687-022-00440-3)
Supplement: Supplementary file 1 — Additional file 1. Full search strategy. [file 41687_2022_440_MOESM1_ESM.docx]

**Additional file 1**

**Appendix 1: search strategy**

PROMIS CAT trauma

| **Database searched** | **via** | **Years of coverage** | **Records** | **Records after duplicates removed** |
| --- | --- | --- | --- | --- |
| Embase | Embase.com | 1971 - Present | 1342 | 1318 |
| Medline ALL | Ovid | 1946 - Present | 818 | 66 |
| Web of Science Core Collection | Web of Knowledge | 1975 - Present | 834 | 147 |
| Cochrane Central Register of Controlled Trials | Wiley | 1992 - Present | 204 | 107 |
| CINAHL | EBSCO | 1982 - Present | 490 | 44 |
| Other sources: Google Scholar top 200 | | | 200 | 69 |
| **Total** | | | **3888** | **1751** |

**Embase.com**

(('patient-reported outcome'/de AND 'information system'/de) OR 'computerized adaptive testing'/de OR (PROMIS OR ((patient-reported-outcome* OR patientreported-outcome*) NEAR/3 (information-system*)) OR computer*-adapt*-test*):ab,ti,kw) **AND** ('injury'/de OR 'limb injury'/exp OR 'fracture'/exp OR 'musculoskeletal injury'/exp OR 'orthopedic surgery'/de OR 'foot surgery'/exp OR 'hand surgery'/exp OR 'fracture treatment'/exp OR 'traumatology'/exp OR (injur* OR trauma* OR fracture* OR orthoped* OR orthopaed* OR surg*):ab,ti,kw)

**Medline (Ovid)**

((Patient Reported Outcome Measures/ AND Information Systems/) OR (PROMIS OR ((patient-reported-outcome* OR patientreported-outcome*) ADJ3 (information-system*)) OR computer*-adapt*-test*).ab,ti,kf.) **AND** ("Wounds and Injuries"/ OR exp Arm Injuries/ OR exp Leg Injuries/ OR exp Fractures, Bone/ OR Orthopedics/ OR Traumatology/ OR (injur* OR trauma* OR fracture* OR orthoped* OR orthopaed* OR surg*).ab,ti,kf.)

**Web of Science**

TS=(((PROMIS OR ((patient-reported-outcome* OR patientreported-outcome*) NEAR/2 (information-system*)) OR computer*-adapt*-test*)) **AND** ((injur* OR trauma* OR fracture* OR orthoped* OR orthopaed* OR surg*)))

**Cochrane Central**

((PROMIS OR ((patient NEXT reported NEXT outcome* OR patientreported NEXT outcome*) NEAR/3 (information NEXT system*)) OR computer* NEXT adapt* NEXT test*):ab,ti,kw) **AND** ((injur* OR trauma* OR fracture* OR orthoped* OR orthopaed* OR surg*):ab,ti,kw)

**Cinahl**

((MH Patient-Reported Outcomes AND MH Information Systems) OR (PROMIS OR ((patient-reported-outcome* OR patientreported-outcome*) N2 (information-system*)) OR computer*-adapt*-test*)) **AND** (MH “Wounds and Injuries” OR MH Arm Injuries+ OR MH Leg Injuries+ OR MH Fractures+ OR MH Orthopedics OR MH Traumatology OR (injur* OR trauma* OR fracture* OR orthoped* OR orthopaed* OR surg*))

**Google Scholar**

PROMIS|“patient reported outcomes measurement information system”|“computerized adaptive testing|tests” injury|injuries|trauma|fracture|fractures|orthopedic|orthopaedic
