# Supplementary material for: There are more things in physical function and pain: a systematic review on physical, mental and social health within the orthopedic fracture population using PROMIS
Source: J Patient Rep Outcomes. 2022 Apr 6;6:34. doi: 10.1186/s41687-022-00440-3 (PMC8986932; doi:10.1186/s41687-022-00440-3)
Supplement: Supplementary file 2 — Additional file 2. STROBE checklist. [file 41687_2022_440_MOESM2_ESM.docx]

**Additional file 2**

Appendix 2. Scores of the included studies on the Strobe checklist

| **First author,**  **Year** | **Level of**  **evidence** | 1a | 1b | 2 | 3 | 4 | 5 | 6a | 6b | 7 | 8 | 9 | 10 | 11 | 12a | 12b | 12c | 12d | 12e | 13a | 13b | 13c | 14a | 14b | 14c | 15 | 16a | 16b | 16c | 17 | 18 | 19 | 20 | 21 | 22 |
| --- | --- | --- | --- | --- | --- | --- | --- | --- | --- | --- | --- | --- | --- | --- | --- | --- | --- | --- | --- | --- | --- | --- | --- | --- | --- | --- | --- | --- | --- | --- | --- | --- | --- | --- | --- |
| Bakhsh et al., 2020 (44) | III | 1 | 1 | 1 | 1 | 0 | 1 | 1 | 1 | 1 | 1 | 1 | 1 | 0 | 1 | 1 | 1 | 1 | 0 | 1 | 1 | 0 | 1 | 1 | 1 | 1 | 1 | 0 | 0 | 1 | 1 | 1 | 1 | 0 | 1 |
| Bhashyam et al., 2018 (45) | IV | 1 | 1 | 1 | 1 | 1 | 1 | 1 | NA | 0 | 1 | 0 | 0 | 0 | 0 | NA | 1 | 1 | 0 | 1 | 1 | 0 | 1 | 1 | 1 | 1 | 1 | NA | NA | 0 | 1 | 1 | 1 | 1 | 1 |
| Bhashyam et al., 2020 (20) | III | 1 | 1 | 1 | 1 | 1 | 1 | 1 | NA | 1 | 1 | 1 | 1 | 1 | 1 | 1 | 1 | 1 | 0 | 1 | 1 | 0 | 1 | 1 | 1 | 1 | 1 | NA | 1 | 1 | 1 | 1 | 1 | 1 | 1 |
| Bozzio et al., 2015 (46) | III | 1 | 1 | 1 | 0 | 1 | 1 | 1 | NA | 0 | 0 | 0 | 1 | 1 | 0 | 0 | 0 | 1 | 0 | 1 | 1 | 0 | 1 | 1 | 1 | 1 | 1 | 0 | 0 | 0 | 1 | 1 | 1 | 1 | 0 |
| Carney et al., 2020 (47) | III | 1 | 1 | 1 | 0 | 0 | 1 | 1 | NA | 1 | 1 | 0 | 1 | 0 | 1 | NA | 1 | 1 | 0 | 1 | 1 | 0 | 1 | 1 | 1 | 1 | 0 | 0 | NA | 1 | 1 | 1 | 1 | 1 | 1 |
| Cavallero et al., 2018 (33) | III | 1 | 1 | 1 | 1 | 1 | 1 | 1 | NA | 1 | 0 | 0 | 0 | 0 | 1 | 1 | 0 | 0 | NA | 0 | NA | 0 | 1 | 0 | 1 | 1 | 0 | 0 | NA | 0 | 1 | 1 | 1 | 1 | 1 |
| Dean et al., 2017 (22) | IV | 1 | 1 | 1 | 1 | 1 | 1 | 1 | NA | 1 | 1 | 0 | 1 | 1 | 1 | 1 | 0 | 0 | 0 | 1 | 1 | 1 | 1 | 1 | 1 | 1 | 1 | 1 | NA | 1 | 1 | 1 | 1 | 0 | 1 |
| Eguia et al., 2020 (48) | III | 0 | 1 | 1 | 1 | 1 | 1 | 1 | 0 | 1 | 1 | 0 | 1 | 1 | 1 | 0 | 0 | 1 | 0 | 1 | 1 | 0 | 1 | 1 | 1 | 1 | 1 | 1 | NA | 0 | 1 | 1 | 1 | 0 | 1 |
| Eguia et al., 2020 (35) | III | 1 | 1 | 1 | 0 | 1 | 1 | 1 | NA | 1 | 1 | 1 | 1 | 1 | 1 | 1 | 0 | 1 | NA | 1 | NA | 0 | 1 | 1 | 1 | 1 | 1 | NA | NA | 1 | 1 | 1 | 1 | 1 | 1 |
| Evans et al., 2020 (49) | III | 1 | 1 | 1 | 1 | 1 | 1 | 1 | 1 | 1 | 1 | 1 | 1 | 1 | 1 | 1 | 1 | 1 | 0 | 1 | 1 | 1 | 1 | 1 | 0 | 1 | 1 | 1 | 1 | 1 | 1 | 1 | 1 | 1 | 1 |
| Fuchs et al., 2015 (50) | III | 1 | 1 | 1 | 0 | 1 | 1 | 1 | 1 | 1 | 1 | 1 | 1 | 1 | 1 | 1 | 1 | 0 | NA | 1 | 1 | 0 | 1 | 1 | 1 | 1 | 1 | NA | NA | 1 | 1 | 1 | 1 | 1 | 1 |
| Gausden et al. 2018 (21) | III | 0 | 1 | 1 | 1 | 1 | 1 | 1 | NA | 1 | 1 | 1 | 1 | 1 | 1 | 1 | 0 | 0 | 0 | 1 | 0 | 0 | 0 | 0 | 1 | 1 | 1 | 1 | NA | 1 | 1 | 1 | 1 | 1 | 1 |
| Gausden et al., 2018 (51) | III | 1 | 1 | 1 | 1 | 1 | 1 | 0 | NA | 0 | 1 | 0 | 1 | 1 | 1 | 1 | 0 | 0 | 0 | 1 | 1 | 0 | 0 | 1 | 0 | 1 | 1 | 1 | NA | 1 | 1 | 1 | 1 | 1 | 1 |
| Gerull et al., 2018 (52) | III | 1 | 1 | 1 | 1 | 1 | 1 | 1 | 1 | 0 | 1 | 1 | 0 | 0 | 1 | 1 | 0 | 0 | 0 | 1 | 0 | 0 | 1 | 1 | 0 | 1 | 1 | 0 | NA | 1 | 1 | 1 | 1 | 1 | 1 |

Abbreviation: NA: not applicable

Appendix 2 continued

| **First author,**  **Year** | **Level of**  **evidence** | 1a | 1b | 2 | 3 | 4 | 5 | 6a | 6b | 7 | 8 | 9 | 10 | 11 | 12a | 12b | 12c | 12d | 12e | 13a | 13b | 13c | 14a | 14b | 14c | 15 | 16a | 16b | 16c | 17 | 18 | 19 | 20 | 21 | 22 |
| --- | --- | --- | --- | --- | --- | --- | --- | --- | --- | --- | --- | --- | --- | --- | --- | --- | --- | --- | --- | --- | --- | --- | --- | --- | --- | --- | --- | --- | --- | --- | --- | --- | --- | --- | --- |
| Gilley et al., 2019 (53) | II | 1 | 1 | 1 | 1 | 1 | 1 | 1 | NA | 0 | 1 | 0 | 1 | 1 | 1 | 1 | 0 | 1 | 0 | 1 | 0 | 0 | 1 | 1 | 1 | 1 | 1 | 1 | NA | 0 | 1 | 0 | 1 | 0 | 1 |
| Glogovac et al., 2020 (54) | III | 1 | 1 | 1 | 0 | 1 | 1 | 1 | NA | 1 | 1 | 1 | 1 | 1 | 1 | 0 | 0 | 0 | 0 | 1 | 0 | 0 | 1 | 1 | 1 | 1 | 0 | NA | NA | 0 | 1 | 1 | 1 | 0 | 1 |
| Jayakumar et al., 2020 (36) | II | 0 | 1 | 1 | 1 | 1 | 1 | 1 | NA | 1 | 1 | 1 | 1 | 0 | 1 | 1 | 1 | 1 | 1 | 1 | 1 | 1 | 1 | 1 | 1 | 1 | 1 | NA | 1 | 1 | 1 | 1 | 1 | 1 | 1 |
| Jayakumar et al., 2019 (55) | IV | 0 | 1 | 1 | 1 | 1 | 1 | 1 | NA | 1 | 1 | 1 | 1 | 0 | 1 | 1 | 0 | 1 | 0 | 1 | 1 | 0 | 1 | 1 | 1 | 1 | 0 | 0 | NA | 1 | 1 | 1 | 1 | 0 | 1 |
| Jayakumar et al., 2019 (56) | II | 0 | 1 | 1 | 1 | 1 | 1 | 1 | NA | 1 | 1 | 1 | 1 | 0 | 1 | 1 | 1 | 1 | 0 | 1 | 1 | 0 | 1 | 1 | 1 | 1 | 0 | 0 | NA | 1 | 1 | 1 | 1 | 1 | 1 |
| Jayakumar et al., 2019 (37) | II | 1 | 1 | 1 | 1 | 1 | 1 | 1 | NA | 1 | 1 | 1 | 1 | 1 | 1 | 1 | 1 | 1 | 1 | 1 | 1 | 0 | 1 | 1 | 1 | 1 | 1 | NA | 1 | 1 | 1 | 1 | 1 | 1 | 1 |
| Jayakumar et al., 2019 (38) | II | 0 | 1 | 1 | 1 | 1 | 1 | 1 | NA | 1 | 1 | 1 | 1 | 1 | 1 | 1 | 1 | 1 | 1 | 1 | 1 | 0 | 1 | 1 | 1 | 1 | 1 | 1 | NA | 0 | 1 | 1 | 1 | 1 | 1 |
| Jayakumar et al., 2019 (39) | II | 0 | 1 | 1 | 1 | 1 | 1 | 1 | NA | 1 | 1 | 1 | 1 | 1 | 1 | 1 | 1 | 1 | 1 | 1 | 1 | 0 | 1 | 1 | 1 | 1 | 0 | 1 | NA | 1 | 1 | 1 | 1 | 1 | 1 |
| Kaat et al., 2017 (57) | III | 1 | 1 | 1 | 1 | 1 | 1 | 1 | NA | 1 | 1 | 1 | 1 | 0 | 1 | 1 | 1 | 1 | 1 | 1 | 1 | 0 | 1 | 1 | 1 | 1 | NA | 0 | NA | 1 | 1 | 1 | 1 | 1 | 1 |
| Kaiser et al., 2020 (58) | III | 0 | 1 | 1 | 1 | 1 | 1 | 1 | 0 | 1 | 1 | 1 | 0 | 1 | 1 | 1 | 0 | 1 | NA | 1 | 1 | 0 | 1 | 1 | 1 | 1 | 1 | NA | NA | 1 | 1 | 1 | 1 | 1 | 1 |
| Kempton et al., 2020 (59) | III | 1 | 1 | 1 | 1 | 1 | 1 | 1 | 1 | 1 | 1 | 1 | 1 | 0 | 1 | 1 | 0 | 1 | 1 | 1 | 0 | 0 | 1 | 1 | 1 | 1 | 1 | 0 | NA | 1 | 1 | 1 | 1 | 1 | 1 |
| Kohring et al., 2020 (60) | III | 1 | 1 | 1 | 1 | 1 | 1 | 1 | 1 | 1 | 1 | 0 | 1 | 1 | 1 | 1 | 0 | 0 | NA | 1 | 0 | 0 | 1 | 0 | 1 | 1 | 1 | NA | NA | 0 | 1 | 1 | 1 | 0 | 0 |
| Metcalf et al., 2020 (61) | III | 1 | 1 | 1 | 1 | 1 | 1 | 1 | NA | 0 | 1 | 1 | 1 | 1 | 1 | 1 | 0 | 1 | 0 | 1 | 1 | 1 | 1 | 0 | 1 | 1 | 0 | NA | NA | 0 | 1 | 1 | 1 | 0 | 1 |
| Minoughan et al., 2017 (62) | II | 0 | 1 | 1 | 1 | 0 | 0 | 1 | NA | 1 | 1 | 1 | 1 | 1 | 1 | 1 | 0 | 0 | 1 | 1 | 1 | 0 | 1 | 1 | 0 | 1 | 1 | 0 | NA | 1 | 1 | 1 | 1 | 1 | 1 |

Abbreviation: NA: not applicable

Appendix 2 continued

| **First author,**  **Year** | **Level of**  **evidence** | 1a | 1b | 2 | 3 | 4 | 5 | 6a | 6b | 7 | 8 | 9 | 10 | 11 | 12a | 12b | 12c | 12d | 12e | 13a | 13b | 13c | 14a | 14b | 14c | 15 | 16a | 16b | 16c | 17 | 18 | 19 | 20 | 21 | 22 |
| --- | --- | --- | --- | --- | --- | --- | --- | --- | --- | --- | --- | --- | --- | --- | --- | --- | --- | --- | --- | --- | --- | --- | --- | --- | --- | --- | --- | --- | --- | --- | --- | --- | --- | --- | --- |
| Morgan et al., 2014 (63) | II | 1 | 1 | 1 | 1 | 0 | 0 | 1 | NA | 1 | 1 | 1 | 1 | 1 | 1 | 1 | 0 | 0 | 1 | 1 | 1 | 0 | 1 | 0 | 1 | 1 | 1 | 1 | NA | 1 | 1 | 1 | 1 | 1 | 1 |
| Ochen et al., 2020 (65) | III | 1 | 1 | 1 | 1 | 1 | 1 | 1 | NA | 1 | 1 | 1 | 1 | 0 | 1 | 1 | 0 | 0 | 1 | 1 | 1 | 0 | 1 | 1 | 1 | 1 | 1 | 1 | 1 | 1 | 1 | 1 | 1 | 1 | 1 |
| Okike et al., 2015 (66) | III | 0 | 1 | 1 | 1 | 0 | 1 | 1 | 1 | 1 | 1 | 1 | 1 | 1 | 1 | 1 | 1 | 1 | 1 | 1 | 1 | 0 | 1 | 1 | 1 | 1 | 1 | 1 | 0 | 1 | 1 | 1 | 1 | 1 | 1 |
| Okoroafor et al., 2018 (67) | II | 1 | 1 | 1 | 1 | 1 | 1 | 1 | 1 | 1 | 1 | 1 | 1 | 1 | 1 | 1 | 1 | 1 | 1 | 1 | 1 | 0 | 1 | 1 | 1 | 0 | 0 | 1 | NA | 1 | 1 | 1 | 1 | 1 | 1 |
| Pet et al., 2020 (40) | IV | 0 | 1 | 1 | 1 | 0 | 1 | 1 | NA | 1 | 1 | 1 | 1 | 0 | 1 | 1 | 0 | 1 | 1 | 1 | 0 | 0 | 1 | 1 | 1 | 1 | 1 | 1 | NA | 1 | 1 | 1 | 1 | 1 | 1 |
| Rothrock et al., 2019 (34) | III | 1 | 1 | 1 | 1 | 1 | 1 | 1 | 1 | 1 | 1 | 1 | 1 | 1 | 1 | 1 | 1 | 1 | 1 | 1 | 1 | 0 | 1 | 1 | 1 | 1 | 1 | 1 | 0 | 1 | 1 | 1 | 1 | 1 | 1 |
| Sandvall et al., 2019 (69) | III | 1 | 1 | 1 | 1 | 1 | 1 | 1 | NA | 1 | 1 | 1 | 1 | 1 | 1 | 1 | 1 | 1 | 0 | 1 | 0 | 1 | 1 | 1 | 1 | 1 | 1 | 1 | NA | 1 | 1 | 1 | 1 | 1 | 1 |
| Shah et al., 2018 (70) | III | 1 | 1 | 1 | 1 | 1 | 1 | 1 | NA | 1 | 1 | 1 | 1 | 1 | 1 | 1 | 1 | 0 | 1 | 1 | 1 | 0 | 1 | 1 | 0 | 1 | 1 | 1 | NA | 1 | 1 | 1 | 1 | 1 | 1 |
| Sharma et al., 2020 (41) | IV | 0 | 1 | 1 | 1 | 0 | 1 | 1 | NA | 1 | 1 | 1 | 1 | 1 | 1 | 1 | 1 | 1 | 0 | 1 | 1 | 0 | 1 | 1 | 1 | 1 | 1 | 1 | NA | 1 | 1 | 1 | 1 | 1 | 1 |
| Smith et al., 2020 (42) | III | 1 | 1 | 1 | 1 | 1 | 1 | 1 | 1 | 1 | 1 | 1 | 1 | 0 | 1 | 0 | 0 | 1 | 0 | 1 | 0 | 0 | 1 | 1 | 1 | 1 | 1 | 0 | 0 | 0 | 1 | 1 | 1 | 1 | 1 |
| Stuart et al., 2015 (71) | III | 1 | 1 | 1 | 1 | 0 | 1 | 1 | 1 | 1 | 1 | 1 | 1 | 1 | 1 | 1 | 0 | 1 | 0 | 1 | 1 | 1 | 0 | 1 | 1 | 1 | 1 | 1 | NA | 0 | 1 | 1 | 1 | 1 | 1 |
| Swarup et al., 2021 (72) | IV | 1 | 1 | 1 | 1 | 1 | 1 | 1 | NA | 1 | 1 | 1 | 1 | 1 | 1 | 1 | 0 | 1 | 0 | 1 | 0 | 0 | 1 | 1 | 1 | 1 | 0 | 0 | NA | 0 | 1 | 1 | 1 | 1 | 1 |
| vd Vliet et al., 2018 (73) | III | 1 | 1 | 1 | 1 | 1 | 1 | 1 | NA | 1 | 1 | 1 | 1 | 1 | 1 | 1 | 0 | 1 | 0 | 1 | 0 | 1 | 1 | 1 | 1 | 1 | 1 | 1 | NA | 1 | 1 | 1 | 1 | 1 | 1 |
| vd Vliet et al., 2019 (74) | III | 1 | 1 | 1 | 1 | 1 | 1 | 1 | NA | 1 | 1 | 1 | 1 | 1 | 1 | 1 | 0 | 1 | 0 | 1 | 1 | 0 | 1 | 1 | 1 | 1 | 1 | 1 | NA | 1 | 1 | 1 | 1 | 1 | 1 |

Abbreviation: NA: not applicable

Appendix 2 continued

| **First author,**  **Year** | **Level of**  **evidence** | 1a | 1b | 2 | 3 | 4 | 5 | 6a | 6b | 7 | 8 | 9 | 10 | 11 | 12a | 12b | 12c | 12d | 12e | 13a | 13b | 13c | 14a | 14b | 14c | 15 | 16a | 16b | 16c | 17 | 18 | 19 | 20 | 21 | 22 |
| --- | --- | --- | --- | --- | --- | --- | --- | --- | --- | --- | --- | --- | --- | --- | --- | --- | --- | --- | --- | --- | --- | --- | --- | --- | --- | --- | --- | --- | --- | --- | --- | --- | --- | --- | --- |
| v Leeuwen et al., 2016 (75) | III | 0 | 1 | 1 | 1 | 0 | 1 | 1 | NA | 1 | 1 | 1 | 1 | 1 | 1 | 1 | 1 | 0 | 0 | 1 | 1 | 0 | 1 | 1 | 1 | 1 | 1 | 1 | NA | 1 | 1 | 1 | 1 | 1 | 1 |
| v Wyngaarden et al. 2021 (76) | III | 1 | 1 | 1 | 1 | 1 | 1 | 1 | NA | 1 | 1 | 1 | 1 | 1 | 1 | 1 | 0 | 0 | 0 | 1 | 0 | 1 | 1 | 1 | 1 | 1 | 1 | 1 | NA | 1 | 1 | 1 | 1 | 1 | 0 |
| Vincent et al., 2018 (78) | III | 1 | 1 | 1 | 1 | 1 | 1 | 1 | NA | 1 | 1 | 1 | 1 | 1 | 1 | 1 | 1 | 1 | 0 | 1 | 1 | 0 | 1 | 1 | 0 | 1 | 1 | 1 | NA | 1 | 1 | 1 | 1 | 1 | 1 |
| Virkus et al., 2018 (79) | III | 1 | 1 | 1 | 1 | 1 | 1 | 1 | 1 | 1 | 1 | 1 | 1 | 0 | 1 | 1 | 0 | 0 | 1 | 1 | 1 | 0 | 1 | 1 | 1 | 1 | 0 | 1 | NA | 1 | 1 | 1 | 1 | 1 | 1 |
| Wilkens et al., 2018 (80) | III | 0 | 1 | 1 | 1 | 1 | 1 | 1 | NA | 1 | 1 | 1 | 1 | 1 | 1 | 1 | 1 | 1 | 0 | 1 | 1 | 0 | 1 | 1 | NA | 1 | 1 | 1 | NA | 1 | 1 | 1 | 1 | 0 | 1 |

Abbreviation: NA: not applicable
