# Supplementary material for: There are more things in physical function and pain: a systematic review on physical, mental and social health within the orthopedic fracture population using PROMIS
Source: J Patient Rep Outcomes. 2022 Apr 6;6:34. doi: 10.1186/s41687-022-00440-3 (PMC8986932; doi:10.1186/s41687-022-00440-3)
Supplement: Supplementary file 3 — Additional file 3. Care guidelines. [file 41687_2022_440_MOESM3_ESM.docx]

**Additional file 3**

Appendix 3. Scores of included studies on the care guidelines.

| **First author,**  **Year** | **Level of**  **evidence** | **1** | **2** | **3a** | **3b** | **3c** | **3d** | **4** | **5a** | **5b** | **5c** | **5d** | **6** | **7** | **8a** | **8b** | **8c** | **8d** | **9a** | **9b** | **9c** | **10a** | **10b** | **10c** | **10d** | **11a** | **11b** | **11c** | **11d** | **12** | **13** |
| --- | --- | --- | --- | --- | --- | --- | --- | --- | --- | --- | --- | --- | --- | --- | --- | --- | --- | --- | --- | --- | --- | --- | --- | --- | --- | --- | --- | --- | --- | --- | --- |
| North et al. 2017 (64) | **IV** | **1** | **1** | **0** | **1** | **1** | **1** | **1** | **1** | **1** | **1** | **1** | **1** | **1** | **1** | **NA** | **NA** | **Na** | **1** | **0** | **0** | **1** | **1** | **1** | **1** | **0** | **1** | **1** | **1** | **0** | **1** |
| Verhiel et al. 2020 (77) | **IV** | **1** | **1** | **0** | **1** | **1** | **1** | **1** | **1** | **1** | **1** | **1** | **1** | **1** | **1** | **NA** | **1** | **0** | **1** | **0** | **1** | **1** | **1** | **NA** | **1** | **1** | **1** | **1** | **1** | **0** | **1** |

Abbreviation: NA: not applicable
