# Supplementary material for: There are more things in physical function and pain: a systematic review on physical, mental and social health within the orthopedic fracture population using PROMIS
Source: J Patient Rep Outcomes. 2022 Apr 6;6:34. doi: 10.1186/s41687-022-00440-3 (PMC8986932; doi:10.1186/s41687-022-00440-3)
Supplement: Supplementary file 4 — Additional file 4. Downs and Black Checklist. [file 41687_2022_440_MOESM4_ESM.docx]

**Additional file 4**

Appendix 4. Scores of the included studies on the Downs and Black Checklist for the Assessment of Methodological Quality of Randomized and Non-Randomized Studies.

| **First author,**  **Year** | **Level of**  **evidence** | 1 | 2 | 3 | 4 | 5 | 6 | 7 | 8 | 9 | 10 | 11 | 12 | 13 | 14 | 15 | 16 | 17 | 18 | 19 | 20 | 21 | 22 | 23 | 24 | 25 | 26 | 27 |
| --- | --- | --- | --- | --- | --- | --- | --- | --- | --- | --- | --- | --- | --- | --- | --- | --- | --- | --- | --- | --- | --- | --- | --- | --- | --- | --- | --- | --- |
| Anthony et al., 2020 (43) | **II** | **1** | **1** | **1** | **1** | **1** | **1** | **1** | **0** | **1** | **1** | **NA** | **NA** | **0** | **0** | **0** | **1** | **1** | **1** | **1** | **1** | **NA** | **NA** | **1** | **0** | **1** | **1** | **1** |
| Ozkan et.al, 2019 (68) | **I** | **1** | **1** | **1** | **1** | **1** | **1** | **1** | **1** | **1** | **1** | **NA** | **NA** | **0** | **1** | **1** | **1** | **1** | **1** | **NA** | **1** | **1** | **1** | **1** | **1** | **1** | **1** |  |

Abbreviation: NA: not applicable
